# Application of the induced membrane technique of tibia using extracorporeal vs. intracorporeal formation of a cement spacer: a retrospective study

**DOI:** 10.1186/s12891-022-05355-0

**Published:** 2022-05-16

**Authors:** Junhao Luo, Fanyu Bo, Jian Wang, Yongwei Wu, Yunhong Ma, Qudong Yin, Yu Liu

**Affiliations:** Orthopaedic Department, Wuxi No. 9 People’s Hospital Affiliated to Soochow University, 999 Liangxi Rd, Wuxi, 214062 Jiangsu China

**Keywords:** Induced membrane technique, Bone defect, Extracorporeal formation, Intracorporeal formation, Injury of induced membrane, PMMA

## Abstract

**Background:**

There were two ways of preparing the cement spacer: intracorporeal and extracorporeal formation. This study aimed to investigate the outcomes of extracorporeal vs. intracorporeal formation of a spacer using the induced membrane technique (IMT) for repairing bone defects of the tibia.

**Methods:**

Sixty-eight patients with tibial defects treated with IMT were analyzed retrospectively. According to the mode of bone cement preparation, patients were divided into intracorporeal and extracorporeal groups (36 vs. 32 respectively). All patients were followed up for 12–48 months (average 18.7 months). The time interval between the first and second stages, the time required to remove the spacer, injury of the IM or bone ends, bone healing and infection control, as well as the functional recovery (Johner—Wruhs scoring), were compared.

**Results:**

There was no significant difference in the preoperative data between the two groups (*P* > 0.05). There was no significant difference in the time interval (12.64 ± 4.41vs. 13.22 ± 4.96 weeks), infection control (26/28 vs. 20/23), bone healing time (7.47 ± 2.13vs. 7.50 ± 2.14 mos), delayed union (2/36 vs. 2/32), nonunion (2/36 vs. 1/32), an excellent or good rate of limb functional recovery (30/36 vs. 26/32) between the intracorporeal and extracorporeal groups (*P* > 0.05). However, the time required to remove (3.97 ± 2.34 min) was longer and the injury of IM or bone ends (28/36) was greater in the intracorporeal group than those in the extracorporeal group (0.56 ± 0.38 min and 1/32, respectively), showing a significant difference (*P* < 0.05).

**Conclusion:**

Both approaches were shown to have similar effects on bone defect repair and infection control. However, intracorporeal formation had advantages in terms of additional stability, while extracorporeal formation had advantages in terms of removal. Therefore, the specific method should be selected according to specific clinical needs. We recommended the extracorporeal or the modified extracorporeal formation in most cases.

**Supplementary Information:**

The online version contains supplementary material available at 10.1186/s12891-022-05355-0.

## Introduction

Bone defects usually originate from high-energy injury, infection, tumor or congenital malformations. Large bone defects cannot heal by themselves, posing a challenge to orthopaedic surgeons [1, 2]. Different surgical techniques have been developed to address this challenge. The three most reported surgical techniques are free vascularized fibular graft, Ilizarov bone transport and the induced membrane technique (IMT) [1]. The free vascularized fibular graft requires excellent microsurgical experience and alters the donor’s healthy limb. The Ilizarov bone transport technique is a slow and painful process with a greater incidence of complications such as nonunion of the docking site and pin-path infection. The IMT involves two stages: the first operative stage includes radical debridement, skin flap repair of the soft tissue if necessary, and insertion of bone cement—polymethylmethacrylate (PMMA) between the bone defect ends; the second operative stage is the removal of the spacer and autologous cancellous bone grafting into the chamber of the induced membrane (IM). The IM can secrete various growth factors including transforming growth factor beta 1 (TGFβ‐1), fibroblast growth factor 2 (FGF‐2), bone morphogenetic protein 2 (BMP‐2) and vascular endothelial growth factor (VEGF) [3–8]. It contains a rich supply of micro-vessels, mesenchymal stem cells (MSC), and osteoprogenitors that can differentiate into mature osteoblasts. Therefore, the IMT is a technically simple and effective method with rapid bone healing for repairing various bone defects, including large segmental defects [3–8]. The use of cement loaded with antibiotics that can be released slowly reduces the infection rate, so the IMT is also an effective treatment for infectious bone defects [9]. Due to a lack of standard approaches toward making the cement spacer, clinical practice is based primarily on personal habits and experience. Patients in this study were divided into an intracorporeal group(in vivo shaping of bone cement spacer) and an extracorporeal group (in vitro shaping of bone cement spacer) according to the mode of preparation of the cement spacer [10]. The approach to the preparation of the spacer involves whether the spacer is difficult to remove, damage risk and integrity of the IM. The integrity of the IM affects its osteogenic activity, which is related to the effect of repairing bone defect to a certain extent. Therefore, it is necessary to know whether the outcomes of the two methods differ. However, it rarely is reported in the literature. The purpose of this study was to retrospectively compare the intraoperative and postoperative outcomes of extracorporeal vs. intracorporeal formation of a spacer using the IMT for repairing bone defects.

## Materials and methods

### Patients

We performed a retrospective review of all patients with a tibial fracture and defects resulting from an open fracture or infection and surgical debridement treated using IMT in our hospital from January 2009 to October 2019. The study was reviewed and approved by the Institutional Review Board of Wuxi No. 9 People’s Hospital (No. WXJY-LY-20010017), and performed in accordance with the ethical standards laid down in the 1964 Declaration of Helsinki and its later amendments. Signed informed consent was obtained from each patient. A total of 80 patients were selected for the present study. Exclusion criteria were: (i) patients who did not receive the second stage of IMT; non-PMMA cement spacer used; (ii) complications from poor stability after the second stage surgery; (iii) the follow-up time after the second stage surgical procedure was less than 12 months; (iv) patients with incomplete follow-up data or poor compliance with treatment. 12 patients (15%) were excluded by these criteria. The remaining 68 patients (85%) met the inclusion criteria and included an intracorporeal group (36 cases) and an extracorporeal group (32 cases) according to the mode of preparation of the spacer.

The average age of patients in the intracorporeal group was 42.08 years (range 13–75 years), and included 22 males and 14 females. There were 8 patients with non-infectious bone defects (comminuted fractures with bone defects), and 28 patients with infectious bone defects (defects with infection). There were 24 patients with segmental bone defects and 12 cases with partial bone defects. 23 patients had an associated injury (tendon, nerve or vascular injury or skin defect). The average length of the bone defects was 5.72 cm (range 2.0–12.0 cm). Bone defect site: 8 cases in the upper segment, 13 cases in the middle segment, and 15 cases in the lower segment of the tibia. Before the IMT, the average times of other surgeries performed was 2.39 per person (range 0–4 surgeries/person) Table 1.

**Table 1:** Comparison of preoperative data between the two groups

Characteristics	Intracorporeal group (*n* = 36)	Extracorporeal group (*n* = 32)	*P-value*
Age(yers)	42.08 ± 17.07	39.78 ± 16.72	0.577
Male	22(61.11%)	19(59.38%)	0.884
Infectious defects	28(77.77%)	23(71.88%)	0.575
Associated injury	23(63.88%)	20(62.50%)	0.906
Segmental defects	24(66.67%)	19(59.38%)	0.618
Defect length(cm)	5.72 ± 2.68	5.22 ± 2.62	0.440
Site of defects(upper/middle/lower)	8/13/15	5/12/15	0.779
Times of surgery	2.39 ± 1.27	2.41 ± 1.36	0.957

The average age of patients in the extracorporeal group was 39.78 years (range 15–71 years), and included 19 males and 13 females. There were 9 patients with non-infectious bone defects (comminuted fractures with bone defects), and 23 patients with infectious bone defects. There were 19 patients with segmental bone defects and 13 with partial bone defects. 20 patients had an associated injury. The average length of the bone defects was 5.22 cm (range 2.0–10.0 cm). Bone defect site: 5 cases in the upper segment, 12 cases in the middle segment, and 15 cases in the lower segment of the tibia. Before the IMT, the patients underwent other surgeries with an average of 2.41 times per person (range: 0–4 times/person) Table 1.

### Surgical methods

In the first stage, thorough bone and soft tissue debridement were performed, including removal of free bone or sclerotic bone without a blood supply, and the medullary cavity was opened. In cases involving infectious bone defects, internal fixations were removed and the wound was washed with hydrogen peroxide, iodophor, and large amounts of saline several times. Pulse irrigation was used until the flushing fluid became clear. For non-infective bone defects, PMMA and internal or external fixation were used, whereas PMMA loaded with the antibiotics (vancomycin or/and gentamicin) and external fixation were used for infectious bone defects.

In the intracorporeal group, the cement was implanted in a dough phase, then shaped to match the size of the bone defect and wrapped at the ends. At the same time, normal saline was used to wash and cool the heating of the cement until it solidified.

**Fig. 1:**
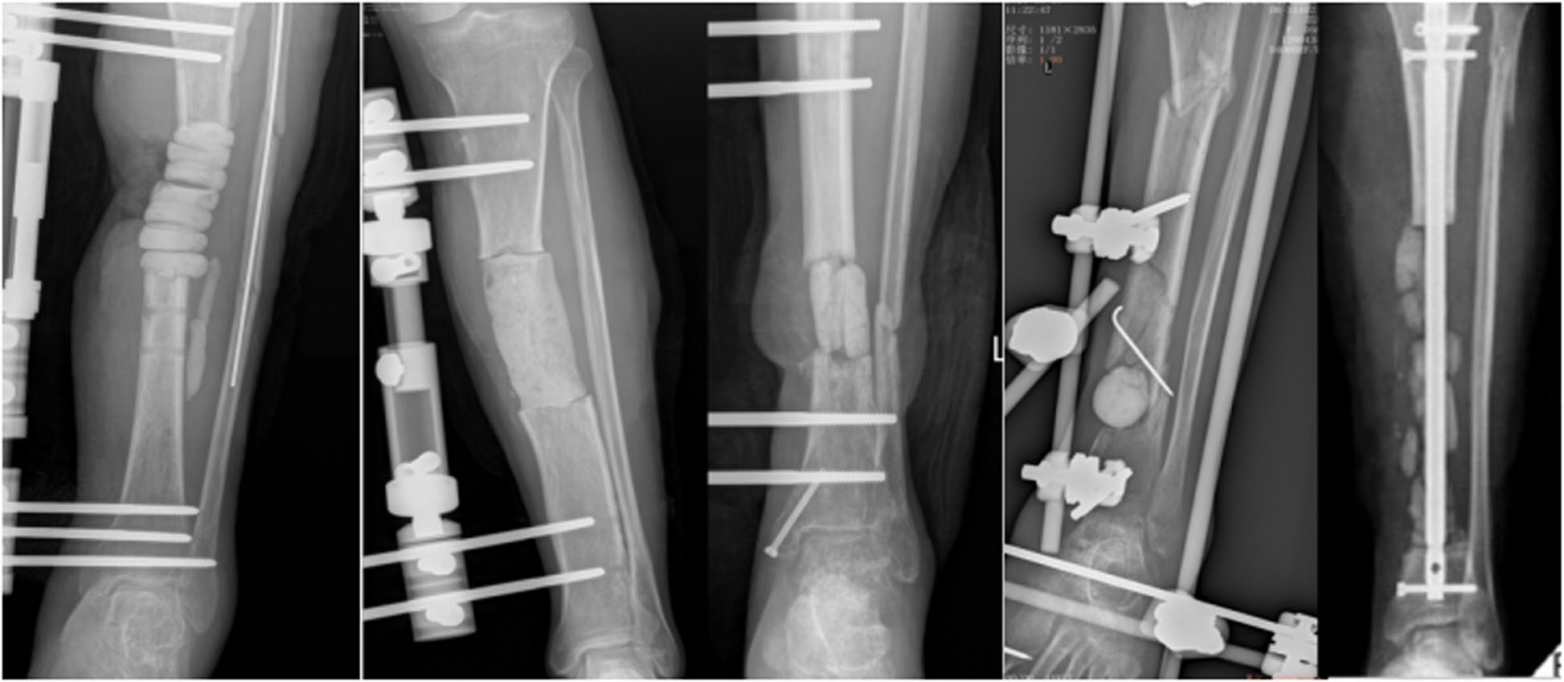
Different shapes of spacer prepared by extracorporeal formation (cylinder, single or multiple blocks, and beads)

In the extracorporeal group, depending upon the size and shape of the bone defect, cylinder, single or multiple blocks or beads of cement were made during the dough phase, and then were inserted into the bone defect site after natural solidification and cooling to room temperature (Fig. 1). In some cases, the multi-column structure pacer was internally fixed with sutures or a thin layer of cement.

The associated skin defect was repaired with a skin flap or myocutaneous flap. For non-infectious bone defects, internal or external fixation was used during the operation, and prophylactic antibiotic therapy was prescribed for 3–5 days after the operation; for infectious bone defects, external fixation was used during the operation, and sensitive or broad-spectrum antibiotics were used intravenously for about 2 weeks following which oral antibiotics were prescribed until 6 weeks after the surgery. For patients with infectious bone defects, the second stage operation of IMT was not performed until the white blood cells, C-reactive protein (CRP), and erythrocyte sedimentation rate (ESR) were reduced to normal or close to the normal levels without clinical symptoms of infection.

In the second stage, the wound was opened through the original surgical approach, the IM was cut longitudinally, the spacer was removed and autologous cancellous bone was grafted into the IM. If the iliac bone graft was insufficient, it was supplemented with no more than one-third of allogeneic bone or artificial bone. Reliable and stable internal fixation including intramedullary nails or locking plates were used. Antibiotics were used for 3–5 days postoperatively.

### Postoperative management

All patients were reexamined every month postoperatively, and every 3 months after bone defect healing. Patients with internal or external fixation began rehabilitation activities 3 days postoperatively and gradually moved adjacent joints. Partial weight-bearing was allowed after callus bridging was confirmed by imaging.

### Evaluation

The time interval between the first and second stages, time required to remove the spacer, injury of the IM or the bone ends, bone healing, infection control and functional recovery were recorded. Injury of the IM or the bone ends was defined as an IM defect area of larger than 1 × 1cm^2^, or an iatrogenic fracture or defects at the end of the bone. Clinical healing was defined as the radiographic presence of bridging bone on 3 of 4 cortices without gross motion or tenderness at the site of the bone defect during a physical examination [11, 12]. In situations where a clear determination could not be made, a CT scan was obtained to confirm union. Nonunion was defined when a minimum of 9 months had elapsed since the fracture or bone defect without visible progressive signs of healing for 3 months. Delayed union was defined as slow healing over 9 months. At the final follow-up, lower limb functional recovery was evaluated using the Johner—Wruhs scoring system [13].

### Statistical analysis

Statistical analyses were conducted using the statistical package SPSS version 17.0 (SPSS Inc., Chicago, IL, USA). For comparison between groups, Student’s t-tests for measurement data of in a normal distribution or Mann–Whitney U test for measurement data of non-normal distribution was used for continuous variables. For categorical variables, the chi-square test was used, whereas Fisher’s exact test was used when the expected counts were < 5. In all analyses, statistical significance was determined by a value of *P* < 0.05.

## Result

### Preoperative data results

There were no significant differences in age, gender, defect type, length and site, associated injury or times of surgeries between the two groups (*P* > 0.05).

### Perioperative period results

**Fig. 2:**
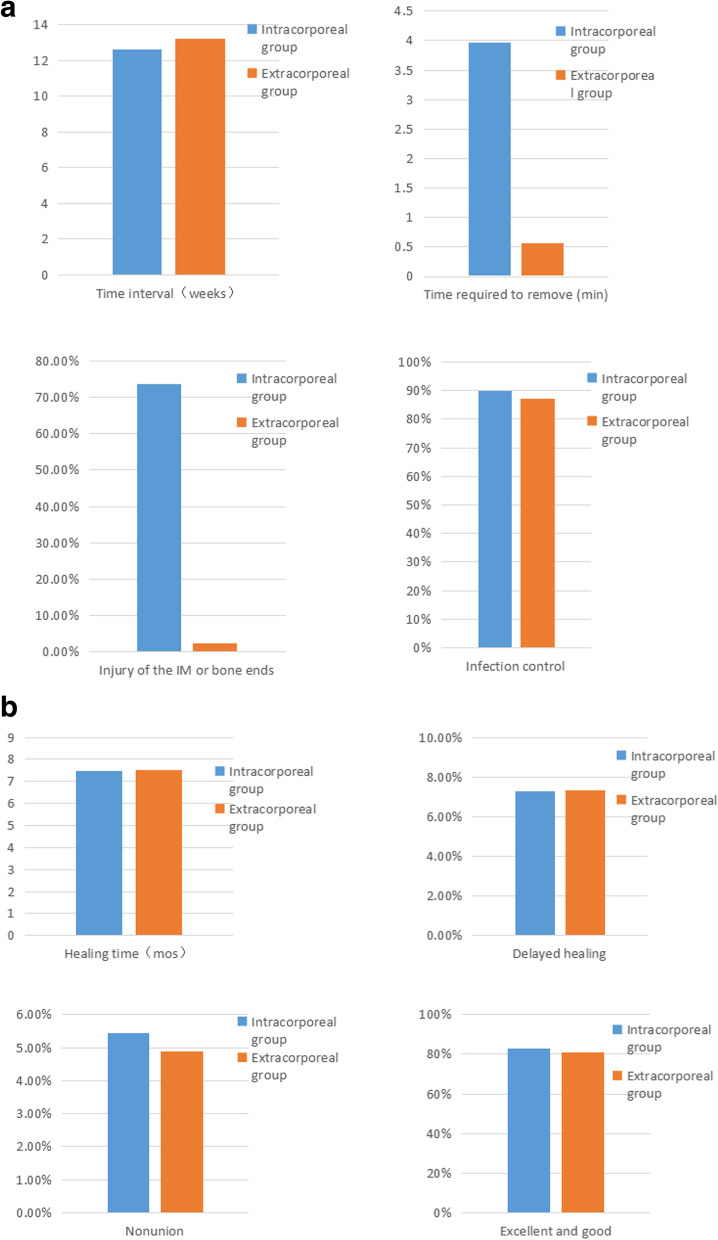
Comparison of the therapeutic effect between the intracorporeal and extracorporeal groups. a The time interval, time requires to remove the spacer, injury of the IM or the bone ends and infection control were compared. b The healing time, delayed healing, nonunion and functional recovery were compared

After the first stage, flap necrosis occurred in 2 cases in the intracorporeal group, and 1 case in the extracorporeal group, resulting in the exposure of the spacer, which needed to be repaired with a skin flap again. There was no significant difference in the time interval between the intracorporeal and extracorporeal groups (12.64 ± 4.41vs. 13.22 ± 4.96 weeks,*P* = 0.611). However, the time required to removal (3.97 ± 2.34 Min) was longer, and the injury of the IM or bone ends ( 28/38) was greater in the intracorporeal group than those (0.56 ± 0.38 min and 1/32) in the extracorporeal group, showing significant difference (*P* < 0.001, each) Table 2 and Fig. 2.

### Complications and follow up

**Fig. 3:**
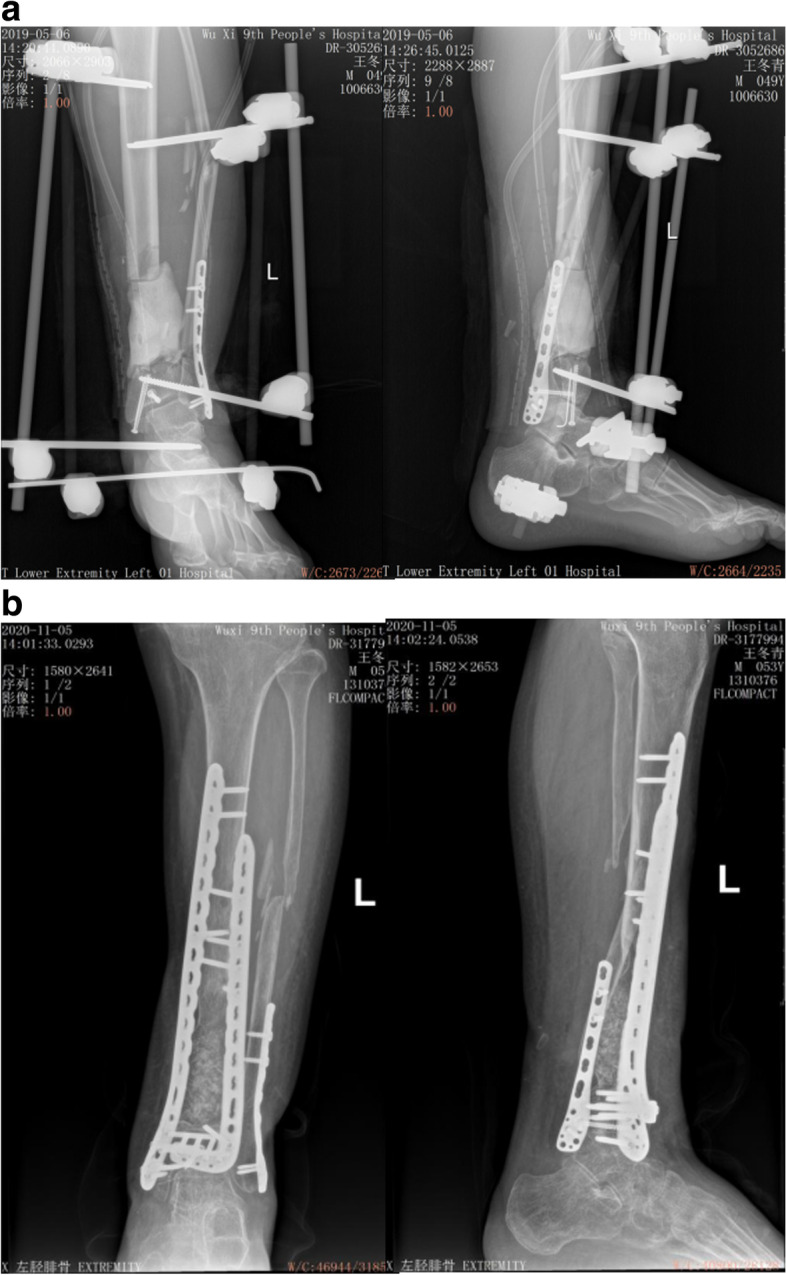
A 49 years-old male patient with segmental defects of the left tibia treated with IMT. a The cement spacer was prepared by extracorporeal formation. B X-rays showed the defects clinically healed in 7 months

**Fig. 4:**
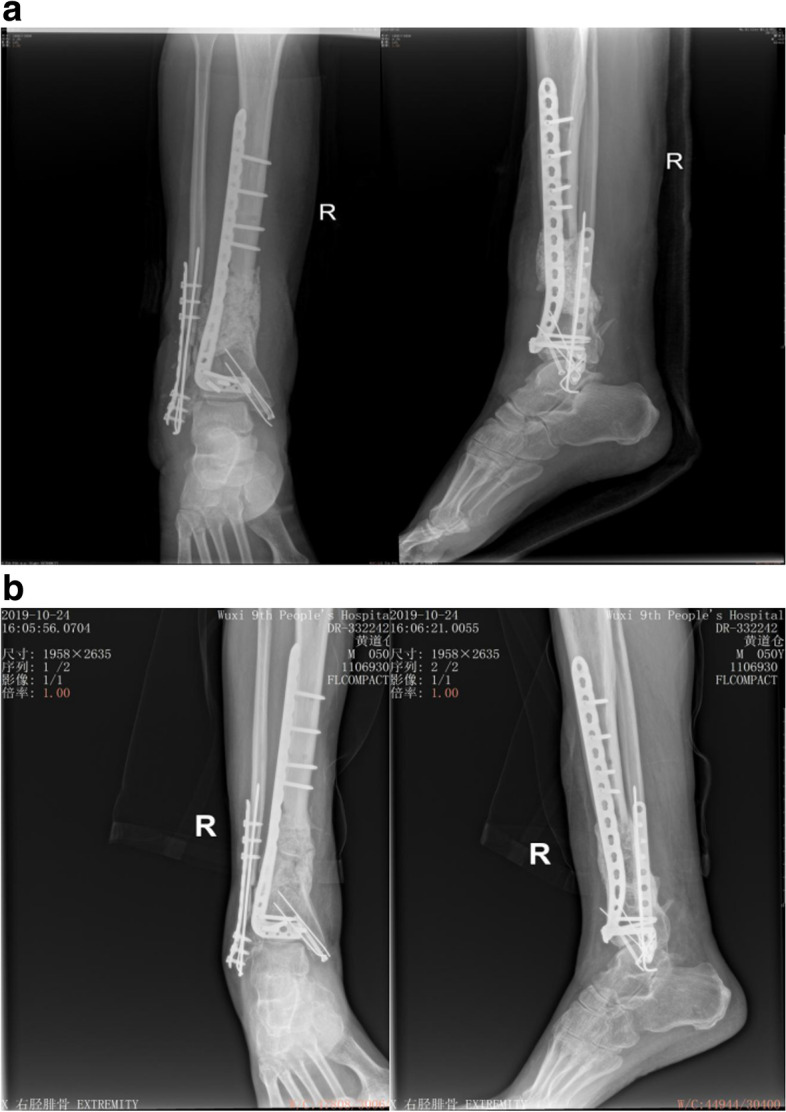
A 47 years-old male patient with segmental defects of the right tibia treated with IMT. a The cement spacer was prepared by intracorporeal formation. b X-rays showed the defects clinically healed in 7 months

All patients were followed up for 12–48 months (average 18.7 months) after the second stage. There were no significant differences in the infection control rate (26/28 vs. 20/23, *P* = 0.647), nonunion (2/38 vs. 1/32, *P* = 0.545), delayed union (2/38 vs. 2/32, *P* = 0.904) and clinical healing time (7.47 ± 2.13vs. 7.50 ± 2.14 mos, *P* = 0.957) between the intracorporeal and extracorporeal groups. 7 cases with bone nonunion and delayed union were mainly related to higher proportion of cortical bone or artificial bone and relatively poor stability of fixation. At the last follow-up, 12 cases were excellent, 18 cases were good, and 6 cases were fair in the intracorporeal group (the excellent or good rate was 83.33%), and 12 cases were excellent, 14 cases were good, and 6 cases were fair (the excellent or good rate was 81.25%) in the extracorporeal group, showing no significant difference between the two groups (*P* = 0.822) Table 2. Typical cases are shown in Fig. 3 and Fig. 4.

**Table 2:** Comparison of outcomes between the two groups

Outcomes	Intracorporeal group (*n* = 36)	Extracorporeal group (*n* = 32)	*P -value*
Time interval(weeks)	12.64 ± 4.41	13.22 ± 4.96	0.611
Time required to remove (min)	3.97 ± 2.34	0.56 ± 0.38	< 0.001
Injury of the IM or bone ends	28(77.77%)	1(2.44%)	< 0.001
Infection control	26(90.24%)	20(86.96%)	0.647
Healing time(mos)	7.47 ± 2.13	7.50 ± 2.14	0.957
Delayed healing	2(5.56%)	2(6.25%)	0.904
Nonunion	2(5.56%)	1(3.12%)	0.545
Excellent and good	30(83.33%)	26(81.25%)	0.822

## Discussion

### Advantages and disadvantages of extracorporeal and intracorporeal formation

This study showed that there were no differences on bone defect repair and infection control. However, there were differences in the time required to removal and the incidence of injury of the IM or bone ends between the two groups.

The classical application of the IMT is intracorporeal formation of a cement spacer. Masquelet and other scholars emphasized that the spacer should wrap 1-2 cm of the ends forming an expanded "collar", in which bone grafting can avoid or significantly reduce the occurrence of bone nonunion [3, 4]; the other advantage of intracorporeal formation is that it can provide additional stability for the bone defect and even restore the stability immediately after surgery. However, there are some disadvantages to intracorporeal formation as well: (1) thermal necrosis of 1–2 mm of the interface bone tissue caused by high heat [3, 4, 10, 14], because a large amount of heat is released, as high as 60°—70° C, in the course of the polymerization of the cement; (2) Because it is tightly connected with the bone interface, and difficult to be removed, biting forceps, bone knife striking or drilling are often used to remove the cement spacer, so the IM and bone ends are easily damaged, destruction of the stability of the original internal fixation, even iatrogenic fracture can occur [3, 10, 14]. Injury of the IM leads to traumatic defects of the IM.

Extracorporeal formation overcomes these disadvantages [3, 10, 14]. In this approach, relatively small single or multiple blocks or beads of cement are implanted into the bone defect after solidification and cooling, avoiding the thermal necrosis of the interface bone tissue. Furthermore, the cement is easy to remove and there is no injury of the IM or bone ends because the cement is not tightly connected to the bone interface and is relatively small. Therefore, it is easy to remove and there were no traumatic defects of the IM and damage to the bone ends and stability of the fixation in these patients. In this study, there were significant differences in terms of the time required for removal and injury of the IM or bone ends between the two groups (*P* < 0.05). In order to increase the stability of bone defects by extracorporeal formation, some authors [10, 15] have reported an improved method of extracorporeal formation of the cement spacer, in which a cylinder is formed and divided into 2–3 blocks in vitro, then implanted it into the bone defect after solidification and cooling, and finally wrapped around the bone ends with a thin layer of cement in vivo during the dough phase to forms a "collar". This improved extracorporeal formation can also provide may also provide additional stability to bone defects, similar to the intracorporeal formation, and was used in our study.

The key of IMT to repair bone defects lies in the IM. The IM has the function of mechanical wrapping and biological osteogenesis, which avoids or reduces the absorption of the grafted cancellous bone and promotes the formation of new bone. Therefore, the bone healing time and healing rate of IMT are better than those of traditional cancellous bone grafting [2]. The integrity and biological activity of the IM are related to the outcomes of bone defect repair. Defects of the IM lead to weakening of the effects of mechanical wrapping and biological osteogenesis of the IM, and might affect the bone defect repair [13]. However, in the study, the differences were not significant in terms of the healing time and healing rate between the two groups, which may be due to smaller defect size of the IM, followed by a small sample size.

It has been reported that the infection control rate of antibiotic-loaded bone cement for infectious defects is 78—100% [16–20]. Walenkamp et al. [17] reported a series of 100 patients with infectious defects treated with gentamicin-loaded PMMA beads who were followed for 5 years. In that sample, the infection control rate was 78% after a single treatment and 92% after two or three treatments. Qiu et al. [20] compared the outcomes of antibiotic-loaded bone cement beads and IMT with intracorporeal formation of the bone cement spacer for post-traumatic osteomyelitis. Their results showed no significant differences in infection control rate, bone healing time or complications between the two groups, but it took more time to remove the spacer in the intracorporeal formation group. In the current study, the infection control rate was 90.20%, the cases with recurrence of infection were related primarily to insufficient debridement; there were no significant differences in the infection control rate, bone healing and functional recovery between the two groups. The above results were consistent with those reported by Qiu et al. [20].

### Choice of indications

In view of both approaches have similar effects on bone defect repair and infection control, intracorporeal formation had advantages in terms of additional stability while extracorporeal formation had advantages in terms of removal. Therefore, intracorporeal formation is especially suitable for bone defects that require additional stability such as severe osteoporosis and/or long segmental bone defects while extracorporeal formation is especially suitable for partial or small segmental bone defects and segmental bone defects fixed with medial and lateral plates and screws where the spacer is difficult to remove [2, 3, 15]. Since bone cement can produce high heat and can kill tumor cells, intracorporeal formation is also especially suitable for defect reconstruction using IMT after resection of bone tumors. However, the modified extracorporeal formation with a “collar”[10, 15] is also suitable for long segmental bone defects as it can provide additional stability to bone defects similar to the intracorporeal formation. Therefore, we recommended the extracorporeal or the modified extracorporeal formation in most cases.

### Limitations of this study

This study only reviewed the treatment outcomes in 68 patients treated with IMT. The sample size is not large enough to determine the effects of the use of different grouping types of internal implant and flap, quantity and quality of bone grafts, etc., there may be bias; in particular the defect size of the IM was not graded and analyzed; the evidence level of retrospective design is low. In addition to the osteogenic activity and integrity of the IM, bone healing is also related to the quality and quantity of bone graft and blood supply. Therefore, more cases and experimental studies are needed to explore the effect of defect size of the IM on the repair effect.

## Conclusions

The results show that the formation of a cement spacer by extracorporeal and intracorporeal methods in conjunction with the use of IMT has similar effects on bone defect repair and infection control. However, intracorporeal formation has advantages in terms of additional stability, while extracorporeal formation has advantages in terms of removal. Therefore, different method should be selected according to different situations. We recommended the extracorporeal or the modified extracorporeal formation in most cases.

## Supplementary Information

**Additional file 1.** (PDF 465 kb)

**Additional file 2.** (MP4 3569 kb)

**Additional file 3.** (PDF 103 kb)

## Data Availability

All data generated or analyzed during this study are included in this published article.
