# Supplementary figures and images for: Application of the induced membrane technique of tibia using extracorporeal vs. intracorporeal formation of a cement spacer: a retrospective study
Source: BMC Musculoskelet Disord. 2022 May 16;23:460. doi: 10.1186/s12891-022-05355-0 (PMC9109293; doi:10.1186/s12891-022-05355-0)

## Intracorporeal formation

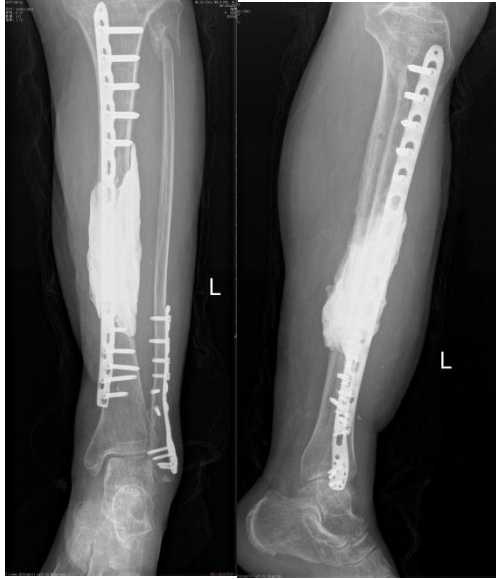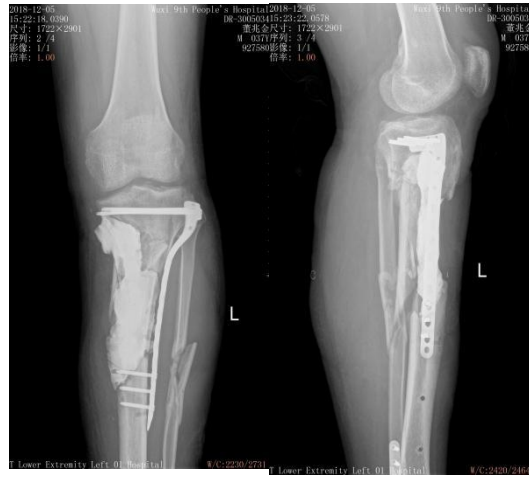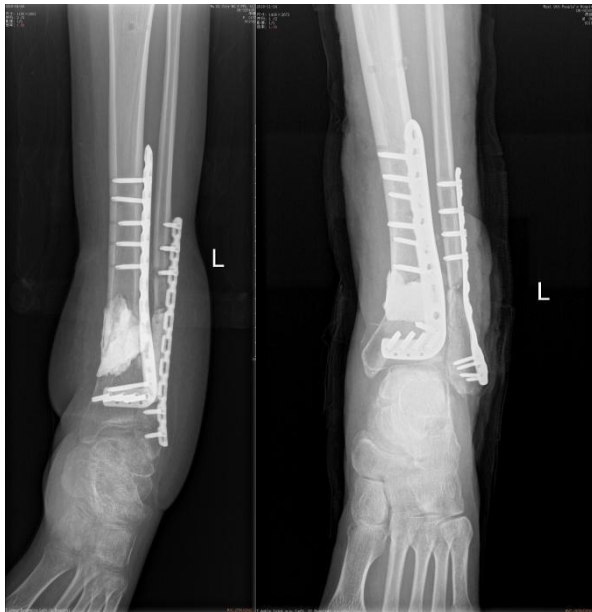

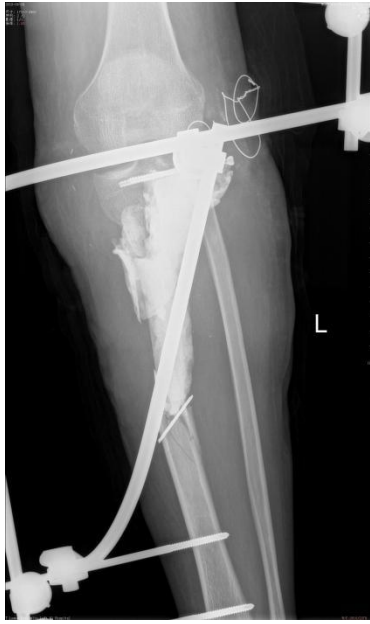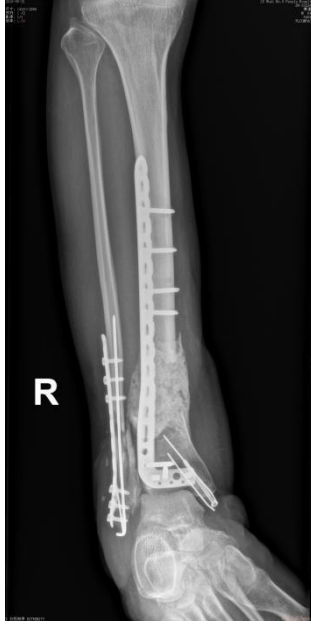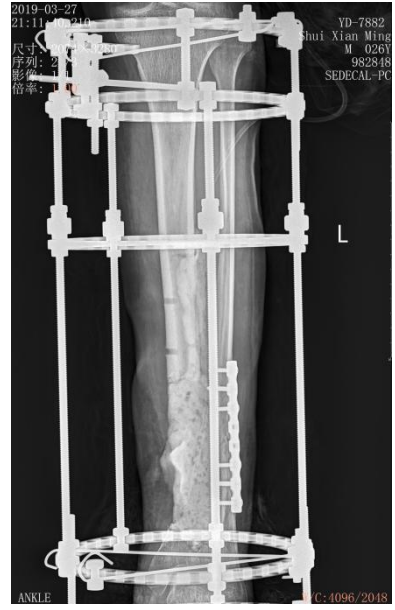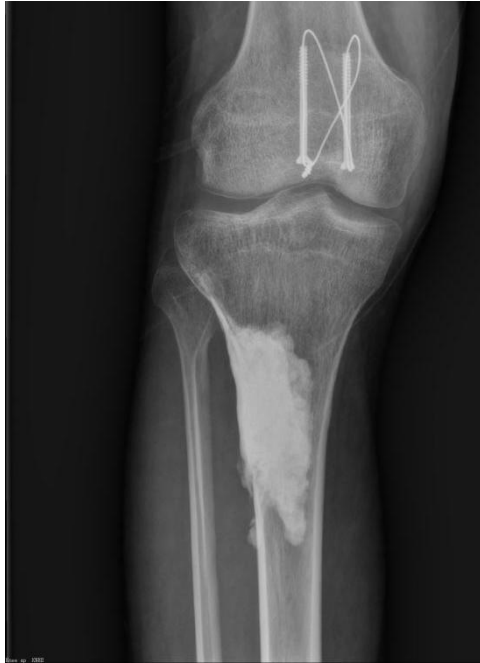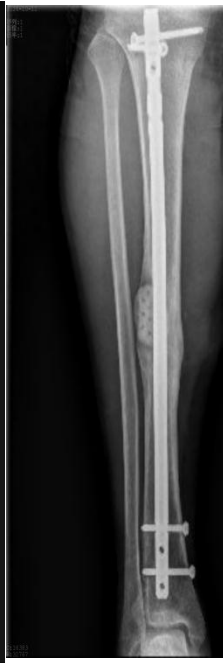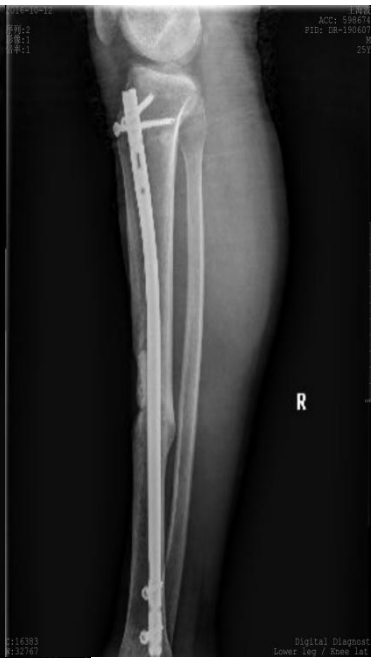

## Extracorporeal formation

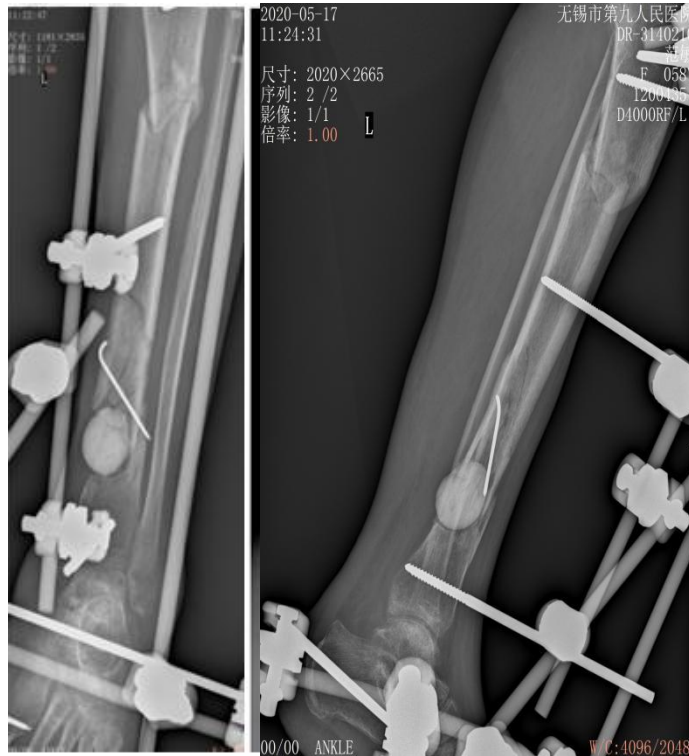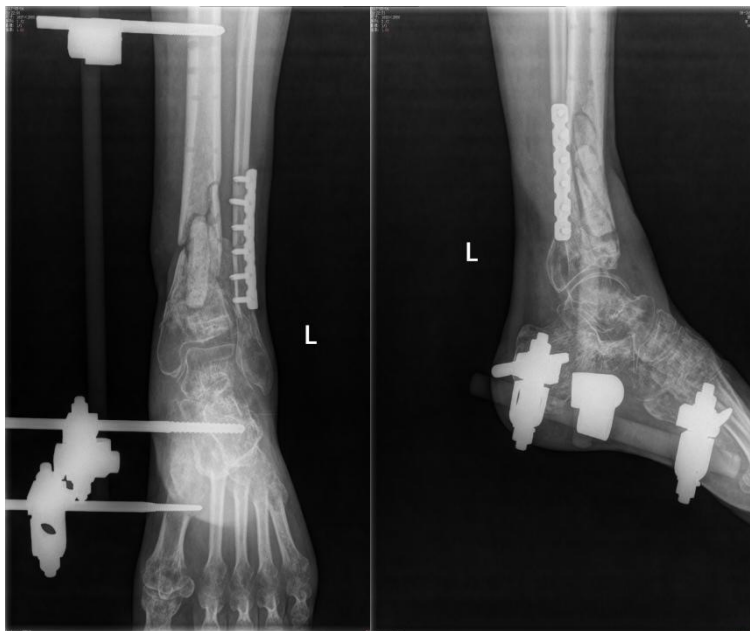

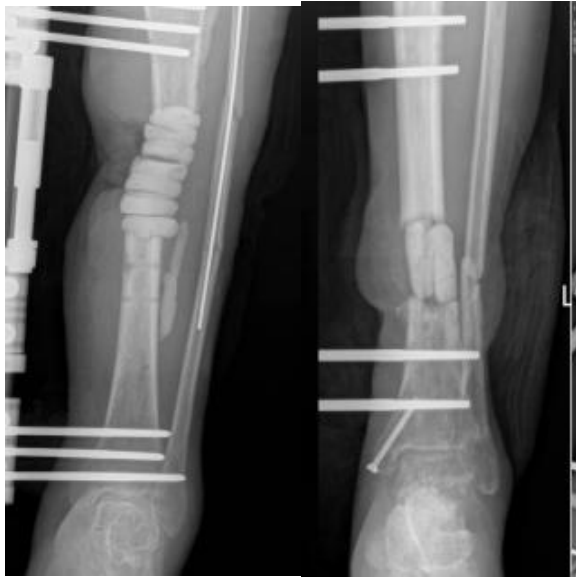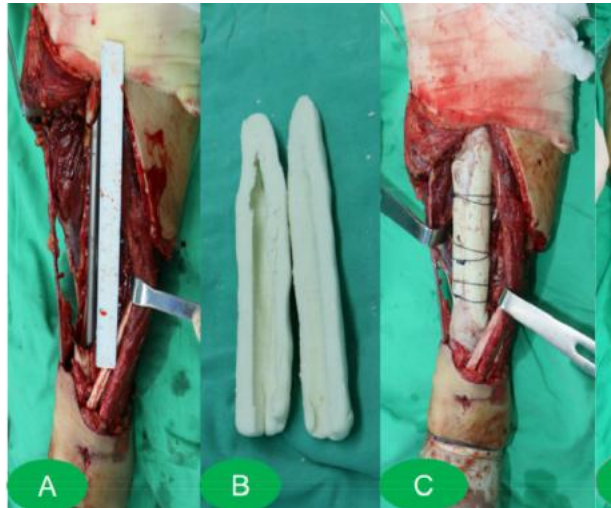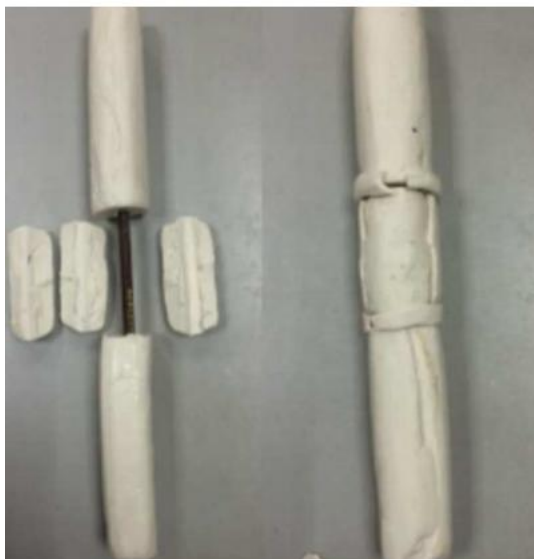

Supplement: Supplementary file 1 — Additional file 1. (PDF 465 kb) [file 12891_2022_5355_MOESM1_ESM.pdf]
