# Supplementary material for: Application of the induced membrane technique of tibia using extracorporeal vs. intracorporeal formation of a cement spacer: a retrospective study
Source: BMC Musculoskelet Disord. 2022 May 16;23:460. doi: 10.1186/s12891-022-05355-0 (PMC9109293; doi:10.1186/s12891-022-05355-0)
Supplement: Supplementary file 3 — Additional file 3. (PDF 103 kb) [file 12891_2022_5355_MOESM3_ESM.pdf]

Induced Membrane Technique: Pearls and Pitfalls

Alain C. Masquelet, MD

Summary: The induced membrane technique for reconstruction of bone defects has proved its effectiveness and is now widely accepted. The 2 stages procedure, implying a cement spacer in the first stage and a huge bone graft in the second stage, responds to rigorous technical details involving each phase of the procedure. Prerequisites for the technique, cement spacer, bone fixation, time between the 2 stages, grafting, and postoperative course are reported and discussed.

Key Words: induced membrane technique, bone loss, Masquelet technique

(*J Orthop Trauma* 2017;31:S36–S38)

Downloaded from http://journals.lww.com/jorthotrauma by BhDMf5ePHKav1zEoum1tQfN4a+kJLhEZgbsIHo4XMi0hCywCX1AWnYQp/IlQrHD3i3D0OdRyi7TvSFl4Cf3VC4/OAVpDDa8K2+Ya6H515kE= on 03/17/2022

INTRODUCTION

The induced membrane (IM) technique has been used for more than 30 years.[1] It has recently gained popularity and is more and more widely accepted all over the world, as a simple and effective technique for reconstruction of segmental bone defects.

Initially described for bone loss resulting from septic non union of the leg, it has been extended to all long bone segments, including the clavicle, whatever the etiology of the bone defect. The IM technique is also indicated in recalcitrant bone non unions without bone loss.

Concept

We know now that the technique is founded on the general concept of the tissular response to an enclosed foreign body which leads to the formation of a surrounding biological active membrane. The IM is well vascularized, it secretes growth factors and contains mesenchymal adult stem cells,[2] which are the conditions for tissue regeneration.

Principle

The technique comprises 2 surgical stages. In the first stage, a cement spacer is placed into the bone defect. A few weeks later, the cement is removed and the cavity formed by the IM is filled up with bone graft. Corticalization of the reconstructed bone segment is acquired in a few months and is independent of the initial size of the defect.

The principle of the technique is very simple and does not need sophisticated expertise even if the biological mechanism remains largely unknown. Until now, fundamental understanding of the biological role of the membrane has not permitted clinical improvement.[3] Nonetheless, the success of the technique, meaning reconstructed bone healing, is dependent on clinical rules which must be strictly respected. Long-term follow-up of more than 100 patients has allowed us to formalize the technical rules and to improve them. The goal of this article is to point out the technical details which are likely to lead to common mistakes and which are, in fact, the keys of the success.

PREREQUISITES FOR THE TECHNIQUE

The IM technique is not a technique for treating bone infection. Some series report failures of bone reconstruction because of recurrent infection and conclude failure of the IM technique itself.[4] This is a great misunderstanding. The IM technique can be used once the infection is definitively eradicated. Sometimes healing of infection implies iterative bone and soft tissue debridement which may require non definitive stabilization. One mistake is to think the vascularization of the membrane allows the healing of infection.

SPACER

We have only the experience of the Polymethylmethacrylate cement as spacer. Other materials have not yet been tried even in experimental studies.

The use of a cement spacer raises the problem of its components and shape and we do not advise the use of antibiotic-loaded cement for 3 reasons:

1. The antibiotic may be inactive on the germ and is likely to increase biological resistance of germs.
2. Some active antibiotics can affect the characteristics of the membrane[5] but we do not know if they modify the biological properties of the membrane.
3. Finally, the absence of recurrent infection with cement without antibiotics is a good sign of healing.

Another common mistake is to think antibiotic-loaded cement is capable of treating bone infection and allowing a less important debridement.

Other components of the cement may play a role in the formation of the membrane.

We always use cement loaded with barium sulfate which is the most irritative radiopaque product.

Accepted for publication July 14, 2017.
From the Reconstructive and Hand Surgery Unit, Department of Orthopedic and Reconstructive Surgery, University Paris VI, Paris, France.
The author reports no conflict of interest.
Supplemental digital content is available for this article. Direct URL citations appear in the printed text and are provided in the HTML and PDF versions of this article on the journal's Web site (www.jorthotrauma.com).
Reprints: Alain C. Masquelet, Department of orthopedic and trauma surgery, hôpital Saint Antoine, 184 rue du Faubourg Saint Antoine, 75012 Paris, France (e-mail: alain-charles.masquelet@aphp.fr).
Copyright © 2017 Wolters Kluwer Health, Inc. All rights reserved.
DOI: 10.1097/BOT.0000000000000979

Copyright © 2017 Wolters Kluwer Health, Inc. Unauthorized reproduction of this article is prohibited.

SHAPE OF THE SPACER

Three shapes are possible to use. The most common is a cylinder which is put into the defect. The spacer should be shaped before its solidification. The spacer should be as big as possible, without compromising the soft tissue and the skin closure. Cement should wrap the bone extremities on 2 or 3 centimeters. During the phase of solidification, soft tissues are protected from heat with a piece of glove, and saline irrigation is refreshing the surrounding tissues.

The main pitfall is to put the cement without wrapping the extremities which is subsequently a factor of non union between the graft and the extremities.

The cylinder spacer is suitable for the reconstruction of solid bones like tibia or femur.

At the second stage, the removal of the cement spacer with an osteotome and a hammer may weaken the bone and the instrumental stabilization for smaller bones like clavicle, forearm bone, proximal and distal humerus, hand, and foot. For the defects located on the smaller bones, we use handmade beads of cement which can be more easily removed at the second stage (see **Figure, Supplemental Digital Content 1**, http://links.lww.com/JOT/A175). Cement beads induce also a membrane and we have not observed difference for graft healing between cylinder spacer and beads.

The third shape looks like a flat pebble. It is used in case of recalcitrant non union without defect. The flat pebble of cement is placed around the site of non union to induce a cavity with membrane for the later graft. The flat pebble is shaped on the operating table and put in place after its solidification to prevent burning the bone (see **Figure, Supplemental Digital Content 2**, http://links.lww.com/JOT/A176).

BONE FIXATION

All fixations are possible, but as far as possible they should be very rigid just after the second stage to promote the revascularization of the graft and more flexible later to enhance corticalization.

Intramedullary nail and external device are suitable. The external device has the advantage to permit progressive weight bearing by removing progressively the rods of the fixator. Plates can also be used, notably at the upper limb, but we do not advise the use of locked screws which provide a very rigid stability, likely to become an obstacle to corticalization of the graft. The pitfall is a too rigid stabilization which is required at the beginning but deleterious later.

WHEN SHOULD THE SECOND STAGE BE PERFORMED?

We think that the only valid rule is the healing of the infection and the perfect healing of the soft tissues, notably when a flap was required. Usually the time between the 2 stages ranges from 6 to 10 weeks. We do not observe difference of bone healing about time between the 2 stages, and we found the same conclusions in literature. A time of several months and even several years[6] does not seem pejorative, probably because stem cells are recruited when doing the graft. Bone graft at the second stage can be considered as a foreign body that is likely to reactivate the biological properties of the membrane, the main role of which is to prevent the resorption of the graft.

REMOVAL OF SPACER

At the second stage, the block of cement spacer must to be exposed directly without dissecting the membrane from the surrounding tissues. The cement is broken with an osteotome and hammer. The cavity is gently cleaned. Two points should be emphasized:

1. The medullar cavities of the extremities must be cleaned and curetted.
2. Small bone chips must be detached from the extremities, maintaining their insertion to the surrounding membrane. Small pieces of graft will be placed between these vascularized chips and the bone extremities, which will avoid a non union (see **Figures, Supplemental Digital Content 3 and 4**, http://links.lww.com/JOT/A177 and http://links.lww.com/JOT/A178).

MATERIAL INSIDE THE MEMBRANE

Gold standard is the cancellous bone harvested from the iliac crests. Cortico cancellous bone is not advocated. Bone block is not suitable because it will not be revascularized by the membrane. Cancellous bone graft must be shaped in little cubes of 1 mm (see **Figure, Supplemental Digital Content 5**, http://links.lww.com/JOT/A179). It can be associated with cancellous bone allograft with a ratio which should not exceed 1/3 because allograft does not contain stem cells and growth factors. The small pieces of cancellous bone should be placed very tightly but not crashed. One main point is to fill the cavity of the membrane in totality. If the graft is not tightly disposed, notably at the upper extremity of the defect, it becomes scattered because of the gravity, when the patient is standing up. We have observed that it is a factor of non union or fracture of the reconstructed segment (see **Figure, Supplemental Digital Content 6**, http://links.lww.com/JOT/A180).

To spare the autograft of the patient, one can use the material harvested by radioimmunoassay procedure[7] which looks like wet sand. This graft, which is routinely harvested at the femur, is too thick to be correctly revacularized by the membrane and should be mixed with cancellous autograft or bone substitute (see **Figure, Supplemental Digital Content 7**, http://links.lww.com/JOT/A181). In our experience, one of the more suitable bone substitutes is the biphasic HA/TCP which is a combination of hydroxiapatite (20%) and beta tricalcium phosphate (80%).[8] This substitute has micro and macroporous structure and has proved osteogenic properties.

One mistake is to think that isolated cancellous allograft or bone substitute inside the membrane is sufficient to obtain a bone consolidation.

Finally, we have observed that it could be deleterious to associate a recombinant growth factor with the graft material.[9] Localized high density of the product and possible effects of competition with secreted growth factors can lead to partial resorption of the graft (see **Figure, Supplemental**

Copyright © 2017 Wolters Kluwer Health, Inc. All rights reserved.

Copyright © 2017 Wolters Kluwer Health, Inc. Unauthorized reproduction of this article is prohibited.

Digital Content 8, http://links.lww.com/JOT/A182). The question remains open to associate recombinant growth factor with bone substitute alone.

In some cases, we routinely used specific procedures:

1. For reconstruction of the tibia, the cement spacer is applied on the fibula, as far as possible (see **Figure**, **Supplemental Digital Content 9**, http://links.lww.com/JOT/A183). At the second stage, the graft is also applied to the fibula and we add an intertibiofibular graft at each extremity of the defect[10] (see **Figure**, **Supplemental Digital Content 6**, http://links.lww.com/JOT/A104).
2. In our opinion, reconstruction of the femur needs an intercalated nonvascularized fibular segment which is placed inside the membrane on the medial aspect of the reconstruction to counteract the bending varus forces. This fibular segment is multiperforated with a small drill to promote its revacularization by the membrane. In all cases, we observed a good integration of the fibular segment without any resorption (see **Figure**, **Supplemental Digital Content 10**, http://links.lww.com/JOT/A184).

CLOSURE

The membrane is closed over the graft with adherent soft tissues. It is mandatory to put drainage inside the cavity of membrane or in the subcutaneous tissue. Without suction drainage, the risk of a hematoma obliging a surgical drainage is very high and can lead to a partial loss of graft (see **Figure**, **Supplemental Digital Content 6**, http://links.lww.com/JOT/A104).

BONE HEALING

Bone consolidation with corticalization is regularly observed within 4 months. This time is not sufficient to allow a total weight bearing without protection for a reconstruction of the lower limb. Whatever the surgical stabilization, recovery of weight bearing should be progressive and is acquired within 6–7 months after the graft.

In conclusion, experience has shown that the IM technique is effective for the reconstruction of long bone defects or recalcitrant non unions. Many failures can be explained by technical mistakes.

REFERENCES

1. Masquelet AC, Fitoussi F, Begue T, et al. Reconstruction of the long bones by the induced membrane and spongy autograft. *Ann Chir Plast Esthet.* 2000;45:346–353.
2. Gruber HE, Ode G, Hoelscher G, et al. Osteogenic, stem cell and molecular characterisation of the human induced membrane from extremity bone defects. *Bone Joint Res.* 2016;5:106–115.
3. Mauffrey C, Giannoudis PV, Conwayu JD, et al. Masquelet technique for the treatment of segmental bone loss: have we made any progress? *Injury*. 2016;47:2051–2052.
4. Morris R, Hossain M, Evans A, et al. Induced membrane technique for treating tibial defects gives mixed results. *Bone Joint J.* 2017;99-B:680–685.
5. Nau C, Seebach C, Trumm A, et al. Alteration of Masquelet's induced membrane characteristics by different kinds of antibiotic enriched bone cement in a critical size defect model in the rat's femur. *Injury*. 2016;47:325–334.
6. Assal M, Stern R. The Masquelet procedure gone awry. *Orthopedics*. 2014;37:e1045–e1048.
7. Stafford PR, Norris BL. Reamer-irrigator-aspirator bone graft bi Masquelet technique for segmental bone defect non-unions: a review 25 cases. *Injury*. 2010;41(suppl 2):S72–S77.
8. Sasaki G, Watanabe Y, Miyamoto W, et al. Induced membrane technique using beta-tricalcium phosphate for reconstruction of femoral and tibial segmental bone loss due to infection: technical tips and preliminary clinical results. *Int Orthop.* 2017 [epub ahead of print]. doi: 10.1007/s00264-017-3503-5.
9. Masquelet AC, Begue T. The concept of induced membrane for reconstruction of long bone defects. *Orthop Clin North Am.* 2010;41:27–37.
10. Fitoussi F, Masquelet AC, Rigal S, et al. Intertibio-fibular graft for traumatic segmental bone defect of the tibia. *Orthop Traumatol Surg Res.* 2012;98:214–219.

Copyright © 2017 Wolters Kluwer Health, Inc. All rights reserved.

Copyright © 2017 Wolters Kluwer Health, Inc. Unauthorized reproduction of this article is prohibited.
